# Impact of viral upper respiratory tract infection on the concentration of nasopharyngeal pneumococcal carriage among Kenyan children

**DOI:** 10.1038/s41598-018-29119-w

**Published:** 2018-07-23

**Authors:** Susan C. Morpeth, Patrick Munywoki, Laura L. Hammitt, Anne Bett, Christian Bottomley, Clayton O. Onyango, David R. Murdoch, D. James Nokes, J. Anthony G. Scott

**Affiliations:** 1 KEMRI-Wellcome Trust Research Programme, Kilifi, 80108 Kenya; 2 Nuffield Department of Medicine, University of Oxford, Oxford, OX3 7FZ United Kingdom; 3 Department of Infectious Disease Epidemiology, the London School of Hygiene and Tropical Medicine, London, WC1E 7HT United Kingdom; 4 Department of International Health, Johns Hopkins Bloomberg School of Public Health, Baltimore, Maryland 21205 USA; 5 Kenya Medical Research Institute (KEMRI), Centre for Global Health Research; KEMRI - CGHR, Kisumu, Kenya; 6 Department of Pathology, University of Otago, Christchurch, New Zealand; 7 Microbiology Unit, Canterbury Health Laboratories, Christchurch, 8011 New Zealand; 8 School of Life Sciences and Zeeman Institute (SBIDER), University of Warwick, Coventry, CV4 7AL United Kingdom; 9 Present Address: Microbiology Laboratory, Middlemore Hospital, Counties Manukau District Health Board, Private Bag 93311, Otahuhu, Auckland 1640 New Zealand

## Abstract

Viral upper respiratory tract infection (URTI) predisposes to bacterial pneumonia possibly by facilitating growth of bacteria such as *Streptococcus pneumoniae* colonising the nasopharynx. We investigated whether viral URTI is temporally associated with an increase in nasopharyngeal pneumococcal concentration. Episodes of symptomatic RSV or rhinovirus URTI among children <5 years were identified from a longitudinal household study in rural Kenya. *lytA* and *alu* PCR were performed on nasopharyngeal samples collected twice-weekly, to measure the pneumococcal concentration adjusted for the concentration of human DNA present. Pneumococcal concentration increased with a fold-change of 3.80 (95%CI 1.95–7.40), with acquisition of RSV or rhinovirus, during 51 URTI episodes among 42 children. In repeated swabs from the baseline period, in the two weeks before URTI developed, within-episode variation was broad; within +/−112-fold range of the geometric mean. We observed only a small increase in nasopharyngeal pneumococcal concentration during RSV or rhinovirus URTI, relative to natural variation. Other factors, such as host response to viral infection, may be more important than nasopharyngeal pneumococcal concentration in determining risk of invasive disease.

## Introduction

Pneumonia is the leading cause of death in children <5 years of age globally, and in Africa^1^. *Streptococcus pneumoniae* is a common cause of childhood pneumonia^2^, and is the foremost cause of vaccine-preventable childhood death in the world^3^. The reservoir of *S*. *pneumoniae* is in the human nasopharynx, particularly the nasopharynges of young children. Nasopharyngeal colonisation is both a necessary precursor to the development of pneumococcal disease^4,5^ and the source of human-to-human transmission.

Viral infections predispose to bacterial, especially pneumococcal, pneumonia. The epidemiology of influenza pandemics^6,7^, and ecological studies of respiratory syncytial virus (RSV) and influenza^8–13^, support this concept. Animal models have confirmed the density and transmissibility of pneumococcal nasopharyngeal carriage increase during viral respiratory infection^14–18^ and basic science experiments demonstrate the upregulation of receptors for bacterial adherence and reduced bacterial clearance during the inflammatory response associated with viral infection^18–24^. Influenza virus, or host responses to influenza virus, have been shown to disperse pneumococci from biofilms, potentiating invasion^25^. In a study in Peru, the risk of nasopharyngeal pneumococcal acquisition increased following acute respiratory illness with influenza or parainfluenza in children <3 years of age^26^, and the quantity of pneumococcal DNA in the nasopharynx was greater during acute respiratory illness than at other time periods^27^.

The association between viral upper respiratory tract infection (URTI) and pneumococcal pneumonia would be explained if viral URTI increased the concentration of pneumococcus in the nasopharynx and in turn this increased the risk of pneumococcal invasion or aspiration, leading to disease. A high concentration of pneumococcus in the nasopharynx has been demonstrated in pneumococcal pneumonia but this does not necessarily mean that the increase in pneumococcal concentration preceded the development of pneumonia^28,29^. To observe dynamic changes in pneumococcal concentration it is necessary to undertake a prospective longitudinal cohort study of nasopharyngeal carriage. Such a study with a pneumonia endpoint would be large, costly and complex. Here we used an existing longitudinal study of household viral transmission^30^ as the framework to examine the concentration of pneumococcal carriage in children aged <5 years before, during and after they acquired an URTI with RSV or rhinovirus.

## Methods

### Sample selection

This study was nested within a longitudinal household study of RSV transmission^30^. Under the parent protocol, 47 households were selected that contained at least one infant and one older sibling and were investigated for a period of 6 months covering one RSV season between December 2009 and June 2010. The households were selected from within the Kilifi Health and Demographic Surveillance System^31^ in rural coastal Kenya, from a population of predominantly subsistence farmers. Nasopharyngeal flocked swab (NPFS) specimens were collected and stored in viral transport medium (VTM), containing gentamicin, twice weekly irrespective of symptoms. While the WHO recommended method for nasopharyngeal pneumococcal detection is a swab stored in skim milk-tryptone-glucose-glycerol (STGG) to enable culture-based methods^32^, it is possible to both detect and quantify pneumococcal DNA in VTM^29^. Multiplex real-time PCR was performed on all samples to detect RSV A/B, adenovirus, rhinovirus and coronaviruses NL63, E229 & OC43^33^. Additionally, five households underwent rhinovirus typing of all samples positive for rhinovirus by sequencing of the VP4-VP2 junction^34^. Rhinovirus was so commonly detected that without typing, separate infection episodes could not be differentiated.

We restricted the present study to episodes of RSV and rhinovirus infections. Influenza virus was not included because the sampling timeframe did not coincide with the influenza season and there were only two influenza episodes that met the selection criteria. Both RSV and rhinovirus have been shown to increase pneumococcal adhesion to human respiratory epithelial cells^19–22,24^; RSV has a strong association with bacterial pneumonia^10–13^; rhinovirus is a common upper respiratory tract virus found in diverse populations of children with respiratory tract symptoms^27,35^.

Samples from children under the age of five years were studied. Periods of viral infection were defined as the detection of RSV or rhinovirus in at least two swabs over a period of 14 days. Only episodes with symptoms of URTI (ie. coryza, cough or sore throat) at some point during the viral infection were considered. A viral infection was defined to begin with the first positive swab for viral infection, with at least one negative preceding swab, and was defined to end at the last positive swab in a series. Periods of viral infection were defined to span missing or virus-negative swabs provided the gap between positive swabs did not exceed 14 days. During the primary study swabs had been defined as positive for respiratory viruses by a real time PCR cycle threshold value of ≤35^36^.

**Figure 1:**
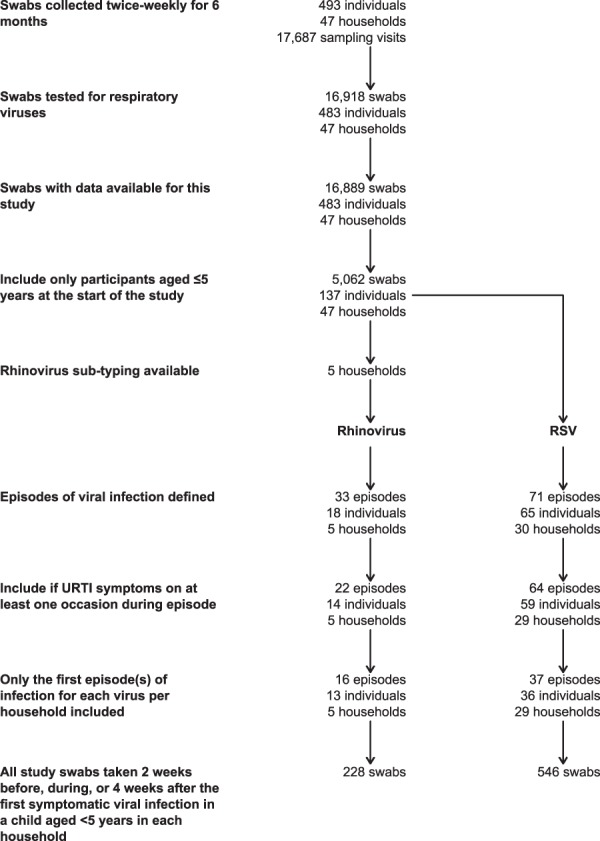
Flowchart describing the selection of viral episodes to examine from the parent study. URTI = upper respiratory tract infection.

Within a single household, one child could have two or more episodes of infection with the same virus, and different children could be infected sequentially throughout the household. However, for this analysis, only the first episode occurring in a child in each household was selected for each virus. If more than one child was infected with the same virus simultaneously, or if a child was the first to be infected in his or her household for both RSV and rhinovirus, or for more than one rhinovirus type, all of those episodes of viral infection were selected. These criteria were applied to ensure that true episodes of viral infection were selected, recurrent episodes were avoided, and that pneumococcal transmission was less likely to have already occurred prior to acquisition of the viral infection, assuming pneumococcus may have been transferred along with the virus from child to child in the household^37^. A study episode was then defined as all available swabs from the two weeks before the viral infection period (the baseline period), all swabs during the viral infection, and swabs from the four weeks after the viral infection. We examined the pneumococcal concentration in all such swabs (Fig. 1).

### Laboratory methods

As part of the parent study, total nucleic acids were extracted from all samples using the MagNA Pure LC (Roche, Indianapolis, USA) automated instrument with the Roche high performance Total Nucleic Acids kit^33^.

A PCR assay for quantification of pneumococcus was performed that detects the pneumococcal autolysin (*lytA*) gene. For *lytA* PCR: forward primer 5′-ACGCAATCTAGCAGATGAAGCA-3′, reverse primer 5′-TCGTGCGTTTTAATTCCAGCT-3′, probe 5′-TGCCGAAAACGCTTGATACAGGGAG-3′ (5′ FAM; 3′ MGB)^38^. Mastermix contained 12.5 μL of Gene Expression Mastermix (Applied Biosystems, Life Technologies, California, USA), 0.5 μL of each of the 10 μM forward and reverse primers and probe, 6 μL of molecular grade water and 5 μL of template DNA per reaction. Quantification standards were *lytA* plasmids (Fast-Track Diagnostics, Luxembourg) diluted 1:10 from 10^8^ copies/mL to 10^2^ copies/mL. Cycling conditions: 95 °C for 10 minutes followed by 40 cycles of 95 °C for 15 seconds and 60 °C for 1 minute were applied on an ABI-7500 real-time PCR instrument (Applied Biosystems, Life Technologies, California, USA).

A PCR for quantification of human DNA was also performed, utilising the multi-copy *alu* gene. For *alu* PCR the forward primer was CAACATAGTGAAACCCCGTCTCT, and the reverse primer was GCCTCAGCCTCCCGAGTAG, each at 10 μM^39^. Mastermix contained 12.5 μL SYBR green (Applied Biosystems, Life Technologies, California, USA), 1.5 μL forward primer, 1.5 μL reverse primer, 4.5 μL molecular grade water and 5 μL of template DNA per reaction. Standards for quantification were made from total human DNA, in µg/mL. Nucleic acid extracts were diluted 1:1000 in molecular grade water. Cycling conditions: 50 °C for 2 minutes, 95 °C for 10 minutes, then 40 cycles of 95 °C for 15 seconds, 59 °C for 1 minute, followed by a slow ramp to a melt of 95 °C for 30 seconds and 59 °C for 15 seconds, on an ABI-7500 (Applied Biosystems, Life Technologies, California, USA).

Pneumococcal concentration in the nasopharynx was first measured as the concentration of pneumococcal DNA per mL of viral transport medium (containing the nasopharyngeal swab sample). Because this is likely to be confounded by variable production and sampling of nasopharyngeal mucous, it was then adjusted by the concentration of human DNA present in the transport medium, to estimate the pneumococcal concentration per μg of human DNA present in the transport medium of the nasopharyngeal swab. Swabs with <1000 copies/mL of *lytA* detected, including swabs with no *lytA* detected, were assigned a value of 500 copies/mL before adjustment for extraction:elution ratio and *alu* concentration on the basis that all such swabs were below the lower limit of detection and linearity of the pneumococcal assay.

### Statistical methods

We summarized the data for each episode of viral URTI studied by calculating the mean natural log pneumococcal concentration (i) before (ii) during and (iii) after viral infection and the difference in these means (before vs during and before vs after). For each comparison, we calculated the mean of the differences over all episodes and obtained a 95% confidence interval using one-sample t-test. To facilitate interpretation, we back-transformed (exponentiated) the mean of the differences to obtain a ratio of geometric means.

A two-sample t-test with unequal variances was used to assess whether the mean difference in log pneumococcal concentration during and before a viral episode were significantly different for RSV and rhinovirus.

To estimate the within and between episode variance, we used episode as the random effect in a one-way random effects model that was fitted to the baseline log pneumococcal concentrations. The between-episode variance estimate includes between-household and between-individual variance since these factors were not included in the model. The model was fitted using the loneway command in Stata.

Because viral infections are known to induce coryza, and increase the volume of nasopharyngeal secretions, we anticipated that some of the observed differences between virus-infected and uninfected children would be attributable to sampled secretion volumes rather than pneumococcal concentration *per se*. We controlled for this by dividing the concentration of pneumococcal DNA in each sample by the concentration of human DNA in the same sample. To illustrate the magnitude of this effect we also present the results of the unadjusted analysis.

The study sample size was chosen to provide 75% power to detect a 3-fold change in pneumococcal concentration between viral infection states^40^.

### Ethical Clearance

The study was approved by the Kenyan National Ethical Review Committee (SSC 1932) and was carried out in accordance with the approved method and all local regulations. In the parent study informed consent was obtained from guardians of all children studied.

### Data Availability

A replication dataset and analysis code is available in the Harvard Dataverse repository (https://dataverse.harvard.edu/dataset.xhtml?persistentId=doi:10.7910/DVN/A0AXNN).

## Results

In all, 17,687 swabs were collected from 47 households over a six-month period. Among these 47 households (five of which had subtyped rhinovirus episodes from the parent study) there were 53 episodes of viral URTI for analysis, among 42 individual children (Fig. 1). In 2 episodes, swabs were unavailable from either the period before viral infection or during viral infection. The remaining 51 episodes were included in the study; 37 episodes of first RSV infection and 14 episodes of first rhinovirus infection. Of 739 selected swabs associated with these episodes, 60 were missing or had been depleted in previous analyses, leaving 679 swabs available for analysis of pneumococcal PCR. Of these, 23/679 swabs had insufficient sample for *alu* qPCR, and two were negative for human DNA by *alu* qPCR, leaving 654 swabs with both *lytA* and *alu* qPCR results.

Of the 42 children involved in the study, 21 (50%) were female, they had a median age of 8.2 months (IQR 4.2 months – 2.9 years) at enrolment into the parent study, and they came from households with a median size of 11 people (IQR 6–16 people).

Swabs “before” one viral URTI could potentially also be “after” another viral URTI; 54 swabs were part of two episodes and four swabs were part of three episodes. Individual plots of pneumococcal concentration before, during and after RSV and rhinovirus infection for each episode can be seen in Supplementary Figure 1. These plots additionally show the samples that were co-infected with another virus; 231/654 (35%) were co-infected. All episodes included coryza as an URTI symptom, and all but five episodes also included cough, none featured a sore throat.

The mean concentration of nasopharyngeal pneumococcus in the baseline periods (swabs collected in the two weeks before viral URTI) was 11.23 natural log copies/μg of human DNA. From the random effects ANOVA, the variation during the baseline period was standard deviation (SD) 1.87 natural log copies/μg between episodes, and within episodes the baseline period variation was SD = 2.36 natural log copies/μg. The frequency distribution of mean log pneumococcal concentrations at baseline was normal, as was the distribution of the residual (within-episode) variation.

The mean fold change in pneumococcal concentration, with and without adjustment for human DNA concentration, for RSV and rhinovirus can be found in Table 1. The fold-change in geometric mean pneumococcal concentration comparing swabs taken during versus before viral infection was 4.63 (95% CI 1.96–10.9) for RSV infections and 2.26 (0.87–5.89) for rhinovirus infections; these were not distinguishable statistically (p = 0.25) and so episodes of infection with both viruses were combined to improve precision in the estimate of effect on pneumococcal concentration (fold-change 3.80, 95% CI 1.95–7.40). The equivalent figure, without adjustment for human DNA concentrations, was 7.47, 95% CI 3.28–17.0.

**Table 1:** Geometric mean fold change in nasopharyngeal pneumococcal concentration with the onset of a symptomatic upper respiratory tract infection (URTI) with RSV or rhinovirus (‘during’-‘before’ viral episode) and with the conclusion of the viral episode (‘after’-‘during’) among children <5 years old.

Changes in mean natural log *lytA* copies/μg human DNA					
Virus	Period of comparison	N of episodes	Fold-change in geometric mean concentration	95% CI	t-test p-value
RSV	during/before	37	4.63	1.96–10.9	0.001
	after/during	37	0.48	0.25–0.91	0.026
Rhinovirus	during/before	14	2.26	0.87–5.89	0.090
	after/during	15	1.33	0.33–5.43	0.668
Combined	during/before	51	3.80	1–95–7.40	<0.001
	after/during	52	0.64	0.35–1.17	0.144
**Changes in mean natural log** ***lytA*** **copies/mL without adjustment for human DNA concentration**					
Combined	during/before	51	7.47	3.28–17.0	<0.001
	after/during	52	0.45	0.20–1.01	0.053

**Figure 2:**
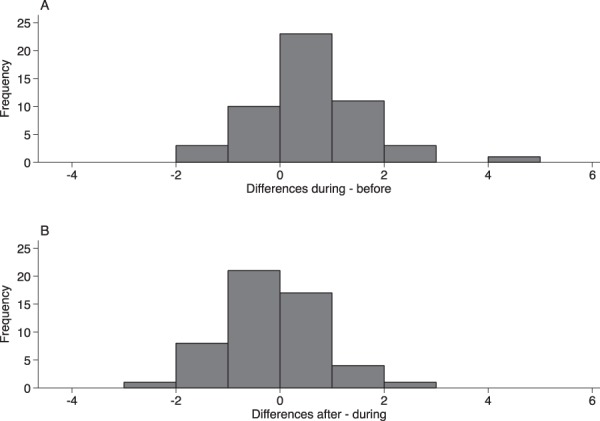
The differences between individual mean nasopharyngeal pneumococcal concentrations in log_10_ *lytA* copies/μg human DNA during and before viral upper respiratory tract infection (URTI), and between pneumococcal concentrations after and during viral URTI. Log_10_ was used instead of natural log in order to aid interpretation.

Pneumococcal concentration fell after viral infection, but this was not statistically significant when episodes of infection with both viruses were combined. For RSV episodes, there was a significant decline, with a fold-change of 0.48 in geometric mean pneumococcal concentration (95% CI 0.25–0.91), between swabs collected during and those collected after the viral URTI. The differences in adjusted pneumococcal concentration during and before viral infection, and after and during viral infection can be seen in Fig. 2.

## Discussion

There is considerable inter and intra-individual heterogeneity in the concentration of pneumococcus in the nasopharynges of children under the age of 5 years in rural Kenya. The within-episode standard deviation in natural log pneumococcal concentration during the baseline period was 2.36, meaning that approximately 95% of the values lie within a 112-fold change (in either direction) of the baseline geometric mean.

The finding of a 4-fold increase in nasopharyngeal pneumococcal concentration, while statistically significant, is therefore very small relative to the variation in baseline within (and between) individuals. Even if the probability of invasion increases with an increase in pneumococcal nasopharyngeal concentration, this 4-fold increase in bacterial concentration with viral infection could only make a small contribution to the likelihood of pneumococcal disease and the likelihood of onward transmission among children with viral infections.

The Peruvian study of childhood pneumococcal carriage density before, during and after acute respiratory illness also found approximately a 4-fold increase in pneumococcal density during acute respiratory illness. A recent study of 4–7 year old children in Wisconsin, USA, found a 10-fold increase in pneumococcal nasopharyngeal density with viral infection, regardless of the presence of symptoms^41^. Neither of these studies assessed changes in nasopharyngeal pneumococcal density at the individual level as we did, and did not look at the magnitude of change relative to the variation within individuals. It appears that the range in pneumococcal density among virus-negative children in Peru, approximately 316–31,600,000, is similarly broad relative to the 4-fold increase in median pneumococcal density with acute respiratory infection. In addition, nasopharyngeal samples were collected monthly in Peru, and four times in a year in Wisconsin, as opposed to twice weekly in our study. Nevertheless, taken together, these three studies suggest that in community settings in very different parts of the world, only small increases in childhood pneumococcal carriage density are found in association with viral URTI.

Most of the work done to establish the association between RSV infection and subsequent pneumococcal disease in humans has been at a population level^8,11–13^, finding correlations between RSV and pneumococcal disease incidence, and attributing ~20% of pneumococcal pneumonia or 3–7% of invasive pneumococcal disease (IPD) to RSV infection. Basic science, using human respiratory cell lines and mouse models, has demonstrated that RSV infection increases pneumococcal concentration, decreases bacterial clearance and promotes pneumococcal adherence. The host inflammatory response to respiratory viral infection may trigger pneumococcal dispersion from biofilms. The modest increase in pneumococcal concentration seen in both this study and the American studies^27,41^, on the context of large fluctuations in density of carriage between and within individuals, may indicate that increasing nasopharyngeal pneumococcal concentration is not a prerequisite to triggering pneumococcal invasion into the bloodstream or translocation into the lungs. Other effects of respiratory viruses may be more important; such as the inflammatory host response, volume of coryza, laryngeal compromise from coughing and possibly changing the expression of pneumococcal genes for phase-change to a more invasive phenotype. We also do not fully understand the relative contributions of pneumococcal serotype and strain. Certainly, some serotypes are more invasive than others, and virulence factors other than capsule also play a role^42,43^.

It is not clear how long the augmented risk period for development of pneumococcal disease is subsequent to viral infection. Some ecological studies have found significant associations between peak viral detection and peak pneumococcal disease with a lag of one week to one month, others have found associations without applying a lag^8–10^. We chose to study samples before, during and up to one month after viral infection. We did not find a significant decline in pneumococcal concentrations after RSV or rhinovirus URTIs in Kenyan children when the viral episodes were combined, but we did note that the decline in concentration was significant after RSV episodes if considered alone. It is possible that the effects of viral infection on pneumococcal concentration are different for different viruses, including other viruses that we did not study, such as influenza or human metapneumovirus. However, the fold-change of approximately a half is still small relative to variation in nasopharyngeal pneumococcal concentration within and between individuals.

It is relatively easy to study co-infection and bacterial density in animal models, but much more difficult to do so in natural human environments because of the frequent sampling required. One of the strengths of our study is the extensive swab collection; 17,687 swabs were collected from 47 households over a six-month period, requiring considerable community co-operation. Our study was therefore ideally positioned to investigate viral-pneumococcal relationships.

Many studies are now using quantitative PCR to assay differences in concentration of pathogens associated with carriage and disease states^28,44,45^, making standardisation of sample collection and laboratory methodology vital for comparisons across studies^5^. Although quantification of pathogens in blood is well established through real-time PCR the estimation of pneumococcal burden in the nasopharynx is complicated by the variable volume of nasal secretions with URTI and the variable mass of specimen collected on a nasopharyngeal swab. To control for these limitations, we used simultaneous quantification of human DNA in the same specimens to standardize all measurements of pneumococcal DNA.

Adjusting nasopharyngeal swab specimens for the concentration of human DNA present in the swab transport medium resulted in a reduction in the effect size measured. This is biologically plausible, because symptomatic viral infection can be expected to increase nasopharyngeal secretions, potentially resulting in a greater volume of sample collected into the swab transport medium. However, the adjustment made little difference to the results, suggesting that the nasopharyngeal flocked swab is efficient at collecting a standard volume of secretion from the posterior nasopharyngeal mucosa.

The swab samples we used had been collected into viral transport medium containing gentamicin, not into STGG as per the WHO standard method^32^. However, because our analysis refers to changes in pneumococcal concentration within individuals over time, using the same methodology, we believe that our results are valid. The Peruvian study^27^ did use STGG as the transport medium, and found a remarkably similar change in pneumococcal concentration with acute respiratory illness. The Wisconsin study^41^ used neither nasopharyngeal swabs nor STGG, but a nose blowing technique and M4RT transport media from Remel Inc.

In our study, rhinovirus sub-typing was available, enabling the study of rhinovirus episodes. Despite high compliance from the nearly 500 occupants of the studied households, there were only 37 first RSV episodes and 14 first rhinovirus episodes from children <5 years old available for analysis. We would have liked to perform the analysis restricted to episodes without viral co-infection, but this would have reduced the data to only three episodes.

We do not know the nature of the relationship between nasopharyngeal pneumococcal concentration and transmission or invasion. In a non-linear system it is possible that a modest rise in pneumococcal concentration due to viral URTI would increase the risk of transmission or of invasion. However, this would only occur if the range of concentrations observed in nature were coincident with the point of greatest dynamic range in the risk function. Without further information on the risk functions with pneumococcal concentration, it is difficult to argue that the relatively small changes in concentration observed in temporal association with viral URTI are important mediators of transmission or of invasive risk. This does not disprove that viral infections are on the causal pathway of pneumococcal disease but rather implies that the link between viral URTI and pneumococcal disease may not be as simple as an increase in pneumococcal density.

In conclusion, there is only a small rise in pneumococcal concentration in the nasopharynges of children who acquire a RSV or rhinovirus URTI. The modest increase in pneumococcal concentration may be a contributing factor to the development of bacterial pneumonia or IPD among children with preceding viral URTI at a population level but other factors, such as the host response to respiratory viral infection, are likely to be more important.

## Electronic supplementary material

Supplementary Figure 1
