# Supplementary material for: Impact of viral upper respiratory tract infection on the concentration of nasopharyngeal pneumococcal carriage among Kenyan children
Source: Sci Rep. 2018 Jul 23;8:11030. doi: 10.1038/s41598-018-29119-w (PMC6056465; doi:10.1038/s41598-018-29119-w)
Supplement: Supplementary file 1 — Supplementary Figure 1 [file 41598_2018_29119_MOESM1_ESM.docx]

­­Impact of viral upper respiratory tract infection on the concentration of nasopharyngeal pneumococcal carriage among Kenyan children

Susan C. Morpeth ^1,2,3*^, Patrick Munywoki ^1^, Laura L. Hammitt ^1,4^, Anne Bett ^1^, Christian Bottomley ^3^, Clayton O. Onyango ^1,5^, David R. Murdoch ^6,7^, D. James Nokes ^1,8^, J. Anthony G. Scott ^1,2,3^

1. KEMRI-Wellcome Trust Research Programme, Kilifi 80108, Kenya. 2. Nuffield Department of Medicine, University of Oxford, Oxford OX3 7FZ, United Kingdom. 3. Department of Infectious Disease Epidemiology, the London School of Hygiene and Tropical Medicine, London WC1E 7HT, United Kingdom. 4. Department of International Health, Johns Hopkins Bloomberg School of Public Health, Baltimore, Maryland 21205, USA. 5. Kenya Medical Research Institute (KEMRI), Centre for Global Health Research; KEMRI - CGHR, Kisumu, Kenya. 6. Department of Pathology, University of Otago, Christchurch, New Zealand. 7. Microbiology Unit, Canterbury Health Laboratories, Christchurch 8011, New Zealand. 8. School of Life Sciences and Zeeman Institute (SBIDER), University of Warwick, Coventry CV4 7AL, United Kingdom.

Running title: Pneumococcal carriage in viral infection

Supplementary Figure 1: Nasopharyngeal pneumococcal concentration before, during and after episodes of symptomatic viral upper respiratory tract infection with RSV or rhinovirus.

Nasopharyngeal pneumococcal concentration in *lytA* copies/μg of human DNA, before, during and after each studied episode of symptomatic upper respiratory tract infection (URTI) with RSV or rhinovirus in children <5 years old. The y-axis of each graph is in log10 scale. The swab numbers on the x-axes are consecutive twice-weekly swabs. Where a swab was not collected or the sample was unavailable, the bar is missing. Where a swab was available but did not have detectable pneumococcus by *lytA* qPCR, it is marked zero. For RSV episodes, swabs collected during the viral infection are marked in red, for rhinovirus these are blue. Swabs two weeks before and four weeks after each viral URTI are black. Bars marked with an asterisk indicate swabs that were co-infected with another respiratory virus by multiplex PCR.
